# Impacts of a National Strategy to Reduce Population Salt Intake in England: Serial Cross Sectional Study

**DOI:** 10.1371/journal.pone.0029836

**Published:** 2012-01-04

**Authors:** Christopher Millett, Anthony A. Laverty, Neophytos Stylianou, Kirsten Bibbins-Domingo, Utz J. Pape

**Affiliations:** 1 Department of Primary Care and Public Health, Imperial College London, London, United Kingdom; 2 Departments of Medicine, Epidemiology and Biostatistics, University of California San Francisco, San Francisco, California, United States of America; University Institute of Social and Preventive Medicine, Switzerland

## Abstract

**Background:**

The UK introduced an ambitious national strategy to reduce population levels of salt intake in 2003. The aim of this study was to evaluate the impact of this strategy on salt intake in England, including potential effects on health inequalities.

**Methods:**

Secondary analysis of data from the Health Survey for England. Our main outcome measure was trends in estimated daily salt intake from 2003–2007, as measured by spot urine. Secondary outcome measures were knowledge of government guidance and voluntary use of salt in food preparation over this time period.

**Results:**

There were significant reductions in salt intake between 2003 and 2007 (−0.175grams per day per year, p<0.001). Intake decreased uniformly across all other groups but remained significantly higher in younger persons, men, ethnic minorities and lower social class groups and those without hypertension in 2007. Awareness of government guidance on salt use was lowest in those groups with the highest intake (semi-skilled manual v professional; 64.9% v 71.0% AOR 0.76 95% CI 0.58–0.99). Self reported use of salt added at the table reduced significantly during the study period (56.5% to 40.2% p<0.001). Respondents from ethnic minority groups remained significantly more likely to add salt during cooking (white 42.8%, black 74.1%, south Asian 88.3%) and those from lower social class groups (unskilled manual 46.6%, professional 35.2%) were more likely to add salt at the table.

**Conclusions:**

The introduction a national salt reduction strategy was associated with uniform but modest reductions in salt intake in England, although it is not clear precisely which aspects of the strategy contributed to this. Knowledge of government guidance was lower and voluntary salt use and total salt intake was higher among occupational and ethnic groups at greatest risk of cardiovascular disease.

## Introduction

Strategies to reduce population levels of salt intake may represent a promising and highly cost effective way of reducing the growing burden of non-communicable disease, particularly in resource constrained settings. Estimates suggest that achieving a 15% reduction in population level salt intake would avert 8.5 million deaths in low and middle income countries over ten years at an annual cost of between 4 to 32 US cents per person. [1] Recent modelling studies projected that reductions in mean daily salt intake in North America and Europe would result in large reductions in cardiovascular disease and considerable cost savings to health care systems. [2], [3], [4], [5] For example, a 3 g/day reduction in salt intake in the US population would decrease the annual number of new cases of CHD by up to 120,000, stroke by 66,000 and myocardial infarction by 99,000 and decrease the annual number of deaths from any cause by up to 92,000. [2]

Accumulating evidence about beneficial impacts of salt reduction on population health has led the World Health Organization to set a global target for adult salt intake (5 g/day) and recommend that national governments institute strategies to achieve this. [6] One of the most ambitious attempts to reduce population salt intake has been undertaken in the UK. [7] This national strategy introduced in 2003 involves voluntary agreements with the food industry to reduce salt content in processed foods, improving food labeling and public awareness campaigns to change personal behavior i.e. reduce salt added during cooking. [8] Recent evidence supports the UK approach of working with industry to reduce salt in processed food over other strategies, such as a salt tax. [3] The UK strategy was strengthened in 2006 when a national target was set to reduce population salt intake to 6 grams by 2010 (this target has now been delayed until 2012) reflecting guidance issued by the UK's Committee on Medical Aspects of Food and Nutrition Policy in 1994 and reiterated by the Scientific Advisory Committee on Nutrition in 2003. [9], [10]

Whole population approaches to prevention may be more likely to reduce social inequalities in health outcomes than interventions targeting high risk groups, although this has been contested. [11], [12] The UK salt reduction strategy might be expected to reduce known inequalities in cardiovascular disease (CVD) for several reasons. First, consumption of fresh fruit and vegetables is lower [13] and consumption of processed foods (which contain approximately 75–80% of dietary salt [14]) may be higher in poorer communities who are likely to benefit most from comprehensive reductions in salt content in food by industry. Second, the strategy involves making greater reductions in salt in cheaper food products which are disproportionately consumed by poorer households. [7] Third, the public awareness campaign component of the strategy was targeted at women from lower social class groups, as these women are regarded as the “gatekeepers” for food purchase and preparation in many UK households. [15] Finally, uniform reductions in salt intake appear to produce greater decreases in blood pressure in older patients and in ethnic minorities who are at higher risk of CVD. [16], [17] The aim of this study was to assess the impact of the strategy on population salt intake, including potential impacts on inequalities, using national survey data from the Health Survey for England.

## Methods

### Sampling and data collection

The data used in this study were derived from the Health Survey for England (HSE). The HSE is an annual survey of people living in private households and is a primary mechanism for monitoring population health in England. The methods of the survey are described in detail elsewhere. [18] Briefly, a two-stage stratified sampling process is employed to obtain an independent, nationally representative sample each year. The core sample from the general population is boosted by sampling from population groups of interest in some years i.e. persons from ethnic minorities in 2004. Interviewers obtain household, socioeconomic and personal details, information on health and illness, and health service use from respondents. Respondents aged above the age of 16 years are then visited separately by a trained nurse. The nurse visit involves anthropometric measurements, blood and urine samples. Respondents are asked for information about prescribed medications.

The percentage of adult respondents (≥16 years) to the HSE who provided a urine sample in each year of the study (2003–2007) was 9.0%, 28.1%, 34.9%, 41.3% and 29.7%.

### Variables

Our main outcome measure was daily salt intake and our secondary outcome measures were knowledge of national guidance on salt intake and self reported information on whether salt is added during cooking or at the dinner table. Knowledge of guidance and use of salt results are presented in Tables 1 and 2. Participants were asked “What do you think are the guidelines for maximum daily salt intake?”, and those correctly identifying 6 grams per day were classified as being aware of the target.

**Table 1:** Awareness of salt guidelines (2007).

		n	% aware of guidance	AOR (95% CI)	% aware of 6 g/d target*	AOR (95% CI)
**Overall**		**6384**	**68.8**	**-**	**33.3**	**-**
**Age**	**16–34**	**1586**	**61.0**	**ref**	**35.6**	**ref**
	35–54	2263	69.2	1.43 (1.25, 1.64)	31.9	0.85 (0.66, 1.09)
	55–74	1911	75.5	1.96 (1.70, 2.27)	32.8	0.93 (0.72, 1.20)
	75+	624	66.4	1.26 (1.04, 1.53)	28.1	0.71 (0.44, 1.15)
**Sex**	**Men**	**2831**	**63.4**	**ref**	**29.3**	**Ref**
	Women	3553	73.0	1.57 (1.41, 1.74)	36.3	1.37 (1.12, 1.68)
**Ethnicity**	**White**	**5780**	**71.1**	**ref**	**32.9**	**Ref**
	South Asian	316	40.8	0.28 (0.22, 0.36)	27.7	0.78 (0.45, 1.36)
	Black	153	53.6	0.47 (0.34, 0.65)	56.3	2.62 (1.29, 5.31)
**Social class**	**I - professional**	**324**	**71.0**	**ref**	**34.6**	**Ref**
	II- managerial technical	1906	74.5	1.19 (0.92, 1.55)	34.3	0.99 (0.64, 1.52)
	III - skilled non-manual	1435	71.8	1.04 (0.80, 1.36)	33.6	0.96 (0.61, 1.50)
	III - skilled manual	996	61.9	0.66 (0.50, 0.87)	29.1	0.78 (0.48, 1.27)
	IV - semi-skilled manual	1072	64.9	0.76 (0.58, 0.99)	32.1	0.90 (0.55, 1.45)
	V - unskilled manual	296	66.2	0.80 (0.57, 1.12)	32.9	0.93 (0.49, 1.74)
**Hypertension**	**No**	**5524**	**68.0**	**ref**	**33.3**	**Ref**
	Yes	860	73.4	1.29 (1.10, 1.52)	33.2	1.00 (0.74, 1.33)

AOR: adjusted odds ratios.

*This based on participants being able to identify the guideline amount as 6 grams per day.

**Table 2:** Self reported voluntary use of salt during cooking and at table.

		2003					2007				
		N	Add salt in cooking		Add salt at table		N	Add salt in cooking		Add salt at table	
			%	AOR (95% CI)	%	AOR (95% CI)		%	AOR (95% CI)	%	AOR (95% CI)
**Overall**		**8621**	**52.0**		**56.5**		**6,281**	**45.8**		**40.2**	
**Age**	**16–34**	**1865**	**48.0**	**ref**	**57.2**	**ref**	**1403**	**44.2**	**ref**	**37.4**	**ref**
	35–54	3186	47.3	0.97 (0.87, 1.09)	55.6	0.94 (0.83, 1.05)	2282	40.1	0.84 (0.74, 0.97)	40.7	1.15 (1.00, 1.32)
	55–74	2732	56.3	1.40 (1.24. 1.57)	56.8	0.98 (0.87, 1.11)	1941	48.0	1.16 (1.01, 1.34)	41.4	1.18 (1.02, 1.36)
	75+	838	64.7	1.98 (1.68, 2.35)	57.0	0.99 (0.84, 1.17)	655	62.4	2.10 (1.74, 2.54)	41.1	1.17 (0.96, 1.41)
**Sex**	**Men**	**3896**	**53.4**	**ref**	**60.9**	**ref**	**2866**	**48.0**	**ref**	**43.2**	**ref**
	Women	4725	50.9	0.90 (0.83, 0.98)	52.9	0.72 (0.66, 0.78)	3415	43.9	0.85 (0.77, 0.94)	37.8	0.80 (0.72, 0.88)
**Ethnicity**	**White**	**8212**	**50.5**	**ref**	**56.9**	**ref**	**5828**	**42.8**	**ref**	**41.2**	**ref**
	South Asian	277	87.4	6.79 (4.75, 9.70)	50.9	0.79 (0.62, 0.78)	291	88.3	10.08 (7.02, 14.47)	28.5	0.57 (0.44, 0.74)
	Black	132	72.7	2.62 (1.78, 3.85)	43.2	0.58 (0.41, 0.82)	162	74.1	3.81 (2.67, 5.44)	24.7	0.47 (0.33, 0.67)
**Social class**	**I - professional**	**452**	**52.7**	**ref**	**51.6**	**ref**	**327**	**45.9**	**ref**	**35.2**	**ref**
	II- managerial technical	2597	49.3	0.87 (0.71, 1.07)	55.7	1.18 (0.97, 1.44)	1964	42.7	0.88 (0.69, 1.11)	37.7	1.12 (0.87, 1.43)
	III - skilled non-manual	2099	51.7	0.96 (0.79, 1.18)	53.3	1.07 (0.88, 1.32)	1478	45.2	0.97 (0.77, 1.24)	38.0	1.13 (0.88, 1.45)
	III - skilled manual	1586	54.4	1.07 (0.87, 1.32)	62.0	1.54 (1.24, 1.90)	1067	48.0	1.09 (0.85, 1.40)	45.2	1.52 (1.17, 1.96)
	IV - semi-skilled manual	1441	53.7	1.04 (0.84, 1.29)	57.3	1.26 (1.02, 1.56)	1119	49.2	1.14 (0.89, 1.46)	42.5	1.36 (1.05, 1.76)
	V - unskilled manual	446	54.5	1.08 (0.83, 1.40)	52.5	1.33 (1.02, 1.73)	326	47.9	1.08 (0.80, 1.47)	46.6	1.61 (1.18, 2.21)
**Hypertension**	**No**	**1808**	**51.3**	**ref**	**57.4**	**ref**	**5406**	**45.1**	**ref**	**41.4**	**ref**
	Yes	6813	54.7	1.15 (1.04, 1.40)	53.2	0.85 (0.76, 0.94)	875	50.1	1.22 (1.06, 1.47)	33.1	0.70 (0.60, 0.82)

AOR: adjusted odds ratios.

Daily salt intake was estimated from a voluntary spot urine test, where urine was collected mid flow in a 100 ml beaker. 10 ml of this urine was then collected in a special collection syringe by the participant, and this 10 ml sample was used for analysis. All samples were processed by the same analyser brand (Olympus AU 640) in different laboratories and all of them underwent internal as well as external quality control and assessment, details of which can be found in the relevant reports. [19] Sodium values in the HSE dataset was transformed into a measure of daily salt intake using the accepted conversion method of 1 gram of salt equalling 17.1 mmol of sodium. [14] Our predictor variables were age, gender, ethnicity, social class and hypertension status. We generated a categorical variable for age; 16–34, 35–54, 55–74 and 75+ years. We categorised ethnicity into white, black and south Asian groups. We used the UK Registrar General's classification of social class as a measure of socio-economic status but due to small numbers we collapsed this into two groups (non-manual, manual) for our analysis of salt intake. Hypertension was defined as having a systolic blood pressure >140 mm Hg or a diastolic blood pressure of >90 mm Hg or taking antihypertensive medications.

### Statistical analysis

We present non-weighted findings as they did not differ markedly to findings weighted to weights produced by the Health Survey for England, designed to correct for selection of households, non response, and population profile. [20] Since the distribution of salt intake was positively skewed, the data was logarithmically transformed before linear regression. Geometric means and their 95% confidence intervals are presented in Table 3. We employ a time-series regression model to estimate the trend of salt intake between 2003 and 2007 with one coefficient for the time-effect. Results from these regressions are presented in Table 4 transformed back into their original scale to allow more meaningful interpretation.

**Table 3:** Trends in geometric mean daily salt intake (grams/day)*.

		Year															
Variable	Category	2003			2004			2005			2006			2007			
		n	Mean	95% CI	n	Mean	95% CI	n	Mean	95% CI	n	Mean	95% CI	n	Mean	95% CI	% change 2003–2007
**Overall**		**1668**	**5.29**	**5.1–5.5**	**2840**	**5.99**	**5.9–6.1**	**4643**	**4.80**	**4.7–4.9**	**8844**	**4.73**	**4.7–4.8**	**4269**	**4.55**	**4.5–4.7**	**−14.0**
**Age**	**16–34**	**388**	**6.56**	**6.1–7.0**	**971**	**7.13**	**6.9–7.4**	**1059**	**6.19**	**5.9–6.4**	**1856**	**6.17**	**6.0–6.4**	**950**	**5.94**	**5.7–6.2**	**−9.5**
	35–54	650	5.26	5.0–5.5	1224	5.94	5.7–6.2	1736	5.05	4.9–5.2	3331	4.91	4.8–5.0	1571	4.77	4.6–4.9	−9.3
	55–74	499	4.72	4.5–5.0	551	4.79	4.5–5.1	1445	3.96	3.8–4.1	2844	4.00	3.9–4.1	1357	3.84	3.7–4.0	−18.6
	75+	131	4.38	4.0–4.8	94	4.05	3.6–4.6	403	3.90	3.7–4.1	813	3.96	3.8–4.1	391	3.60	3.4–3.8	−17.8
**Sex**	**Men**	**735**	**6.10**	**5.9–6.4**	**1279**	**6.36**	**6.2–6.6**	**2085**	**5.50**	**5.4–5.7**	**3977**	**5.41**	**5.3–5.5**	**1926**	**5.16**	**5.0–5.3**	**−15.4**
	Women	933	4.73	4.5–4.9	1561	5.70	5.5–5.9	2558	4.26	4.1–4.4	4667	4.24	4.2–4.3	2343	4.12	4.0–4.2	−12.9
**Ethnicity**	**White**	**1573**	**5.21**	**5.0–5.4**	**638**	**4.81**	**4.6–5.1**	**4397**	**4.74**	**4.6–4.8**	**8215**	**4.63**	**4.6–4.7**	**3902**	**4.47**	**4.4–4.7**	**−14.2**
	South Asian	50	5.46	4.6–6.5	1245	6.14	5.9–6.4	121	6.15	5.5–6.9	320	6.44	6.0–6.9	186	5.58	5.1–6.1	+2.2
	Black	26	8.44	7.0–10.1	650	6.89	6.6–7.2	45	6.30	5.1–7.7	168	6.52	5.9–7.2	90	5.52	4.8–6.4	−34.6
**Social class**	**Non Manual**	**944**	**5.04**	**4.8–5.3**	**1337**	**5.91**	**5.7–6.1**	**2783**	**4.52**	**4.4–4.6**	**5219**	**4.42**	**4.3–4.5**	**2510**	**4.32**	**4.2–4.4**	**−14.3**
	Manual	661	5.61	5.3–5.9	1066	6.02	5.8–6.3	1675	5.24	5.1–5.4	3260	5.15	5.0–5.3	1562	4.87	4.7–5.0	−13.2
**Hypertension**	**Yes**	**276**	**4.84**	**4.5–5.2**	**395**	**5.00**	**4.7–5.3**	**815**	**4.30**	**4.1–4.5**	**1595**	**4.21**	**4.1–4.3**	**3477**	**4.09**	**3.9–4.3**	**−15.5**
	No	1072	5.40	5.2–5.6	1903	6.30	6.1–6.4	2938	4.90	4.8–5.0	5957	4.83	4.8–4.9	792	4.67	4.6–4.8	−13.5

*Note that these values come from a spot urine test, and so do not reflect intake over whole 24 hour periods.

**Table 4:** Trends in salt intake from linear time-series regression models.

Model	Category	Adjusted geometric mean in 2003 (g/d)	Regression coefficient for slope (relative to reference category)	Overall regression coefficient for slope	95% Lower CI (Log scale)	95% Upper CI (Log scale)	Reduction in grams/day per year	P value for differences between groups*
**Age**	**16–34**	**6.77**	**ref**	**−0.036**	**−0.055**	**−0.018**	**−0.242**	**<0.001**
	35–54	5.24	0.009	−0.030	−0.013	0.031	−0.155	0.418
	55–74	4.74	−0.003	−0.040	−0.026	0.020	−0.186	0.788
	75+	4.36	0.002	−0.040	−0.032	0.036	−0.171	0.903
**Sex**	**Men**	**6.05**	**ref**	**−0.038**	**−0.050**	**−0.018**	**−0.226**	**<0.001**
	Women	4.66	0.009	−0.030	−0.007	0.025	−0.138	0.265
**Ethnicity**	**White**	**5.21**	**ref**	**−0.034**	**−0.043**	**−0.025**	**−0.173**	**<0.001**
	South Asian	5.14	0.016	−0.010	−0.016	0.047	−0.051	0.333
	Black	8.09	−0.016	−0.050	−0.057	0.026	−0.394	0.465
**Social Class**	**Non Manual**	**5.01**	**ref**	**−0.040**	**−0.048**	**−0.026**	**−0.197**	**<0.001**
	Manual	5.60	0.009	−0.030	−0.030	0.010	−0.165	0.272
**Hypertension**	**Normotensive**	**5.38**	**ref**	**−0.040**	**−0.046**	**−0.027**	**−0.211**	**<0.001**
	Hypertensive	4.78	0.014	−0.030	−0.007	0.034	−0.141	0.186
**Year overall**		**5.25**	**−0.034**	**−0.034**	**−0.043**	**−0.025**	**−0.175**	**<0.001**

*p values for reference categories reflect differences of the trend from zero, while all other values reflect differences from the reference group trend.

For the regression baseline effects were included for the factors age, gender, social class, ethnicity and hypertension. To disentangle time-dependent effects of the factors, we built additional models for each factor containing all baseline effects, the time-dependent interaction effect with the selected factor. Each model also included a separate term for each ethnic group to take account of the varying trends and group composition over time.

We abstain from a full model including all time-dependent interactions as this model would be complex with many parameters. Thus, the models presented in Table 4 are of the trends for the results presented by each dependent variable but controlling for other variables as baseline effects. All statistical analysis was performed using Stata 10.

## Results

### 1. Knowledge of national guidance on daily salt intake in 2007

Seven in ten (68.8%) respondents were aware that the government had issued guidance advising that they should restrict their salt intake (Table 1). Awareness was lower in the youngest aged group (16–34 years) than the older age groups. Women were significantly more likely to be aware of the guidance than men (73.0% v 63.4%; AOR 1.57 95% CI 1.41, 1.74) Black and south Asian respondents were significantly less likely to be aware of the guidance than white respondents (black 53.6% v south Asian 40.8% v white71.1%). Respondents from manual social class groups were significantly less likely to be aware than non-manual groups. One third of respondents (33.3%) were able to state that the recommended daily level of salt intake was 6 grams. Women were more likely to be aware of this recommendation than men and black respondents were more likely to be aware of this recommendation than white respondents (56.3% vs. 32.9%; AOR 2.62 95% CI 1.29, 5.31).

### 2. Voluntary salt use in cooking and at the table

The percentage of respondents who reported adding salt during cooking decreased significantly from 52.0% in 2003 to 45.8% in 2007 (p<0.001 – Table 2). Older respondents (≥55 years), men, respondents from ethnic minority groups and those with hypertension were significantly more likely to add salt during cooking in 2003 and 2007 (Table 2). There was no difference in the percentage reporting adding salt during cooking by social class grouping in either year.

The percentage of respondents who reported adding salt to food at the table decreased significantly from 56.5% in 2003 to 40.2% in 2007 (p<0.001). Men and respondents from lower social class groups were significantly more likely to add salt at the table than women and those from higher social class groups respectively in 2003 and 2007. Respondents from ethnic minority groups and those with hypertension were significantly less likely to add salt at the table than the white group and those without hypertension respectively in both years.

### 3. Trends in daily salt intake from excretion data, 2003–2007

Mean salt intake decreased significantly across all groups between 2003 and 2007 (Tables 3 and Table 4). Findings from our linear time-series regression models suggest that these reductions were uniform between age, sex, social class and ethnic groups and by hypertension status (Table 4). For example, salt intake in white respondents as the reference group declined significantly (0.173 grams/day p<0.001) every year between 2003 and 2007 and declines in black respondents were not found to be significantly different 0.394 grams/day, p value for difference = 0.465), and the same was true of South Asians. Similarly, there is no significant difference in reductions in salt intake between manual and non-manual workers (p value for difference = 0.272). These results are robust against using a more differentiated set of social class. Consequentially, initial differences in salt intake between groups evident in 2003, i.e. higher intake in younger people, men, ethnic minorities and lower social class groups and respondents without hypertension, persisted in 2007 (Table 3).

## Discussion

### Main findings

Our findings suggest that the UK's salt reduction strategy has resulted in significant but modest reductions in population level salt intake. These reductions were broadly uniform across groups which meant that intake remained higher in younger people, men, ethnic minorities and lower social class groups. Voluntary use of salt during food preparation reduced significantly during the study period. Respondents from ethnic minority groups remained much more likely to add salt during cooking and those from lower social class groups remained more likely to add salt at the table. The higher intakes in these groups continued despite being targeted within the campaign and means that they remain at elevated risk of cardiovascular diseases.

Our findings that salt intake is decreasing by 0.175 grams/day per year are consistent with the limited data available from 24 hour urine surveys conducted in the UK to monitor this strategy which found that salt intake decreased by 0.2 grams/day per year (from 9.0 to 8.6 grams) between 2006 and 2008. [21] The higher salt intake in men identified in our study may not only reflect greater energy consumption but lower knowledge of government guidance and greater voluntary use of salt in food preparation. Consistent with previous research we found higher salt intake in young people and ethnic minorities and increased voluntary salt use in lower socio-economic groups. [21], [22], [23]

Precise data on the reductions of salt across the whole diet is difficult to assemble, but the Federation of Bakers claim that UK bakers have reduced the amount of salt in bread by 10% in the last three years, [24] while the Association of Cereal Food Manufacturers in 2007 claimed to have reduced salt levels by 38% from 1998 levels. [25] A review commissioned by the Food Standards Agency found that there were reductions of up to 70% were found in some foods, and highlighted large improvements in areas where different manufacturers worked together under trade or umbrella organisations. [26] The National Diet and Nutrition Survey remains an important part of policy for measuring nutritional status in the UK population, and is due to report on salt intake in 2012. [27]

### Strengths and limitations

Our study had a number of strengths and limitations. The HSE is a representative national survey and a primary mechanism for monitoring population health in England. Only one in three respondents provided a urine sample in most years of the study period. However, the characteristics of respondents who provided a sample did not differ from those that did not in terms age, sex, ethnicity or social class. Comparing outcomes across time using cross-sectional surveys may introduce bias, given that there may be systematic differences in respondents sampled in the different survey years. Estimates of salt intake were derived from spot rather than the “gold standard” 24 hour urine samples. Spot urines are not suited to measuring intake in individuals but available information suggests that this method is appropriate for population monitoring as measurement bias as they are, is likely to be consistent over time and between groups and are biased toward underestimating salt intake. [23], [28] Data from comparisons of spot and 24 hour urine sampling report the non-parametric correlation Spearman coefficients between these two methods to be between 0.42 and 0.50 for sodium, and note that spot urine tests follow the same patterns as 24 hour sampling. Taken together, the authors suggest that spot urine tests can be used to differentiate between groups in the population in a similar way to 24 hour samples. [28]

As the decision to provide a urine sample was voluntary there may be some degree in self selection. The proportion of the overall population providing a sample in each year ranged from 9.0% to 41.3% which may have affected the results presented here. However, analyses using the weights provided by the Health Survey for England to correct for these issues did not influence the results found.

The modest reductions in population level salt intake identified in our study coincided with improvements in blood pressure levels. Data from the Health Survey for England have shown steady improvements in controlling blood pressure from 1994 [29] with particularly large improvements between 2003 and 2006. However, we were unable to isolate the impacts of the salt reduction strategy from other primary and secondary prevention interventions, including the provision of financial incentives to general practitioners, to improvements in blood pressure control over the study period [19], [30], [31].

The data used for this study are now four years old and our findings may not reflect current public knowledge about recommended levels of salt intake or patterns in intake. As the data is limited to 2003 after the intervention, we cannot measure the trend of salt intake prior to the reduction. Therefore, reduced salt intake could be due to a previous trend or a parallel intervention. Based on other evidence however, salt intake was constant before the intervention [23]. We were also unable to measure awareness of salt guidelines prior to 2007, as this year was the first year for which this data was collected.

### Implications for policy

Implementation of a national salt reduction strategy in the UK has been associated with modest reductions in population levels of salt intake [32]. The Food Standards Agency reported that as of 2009 there were over 90 organisations committed to reducing salt in their products ranging from manufacturers, retailers, trade associations and the catering sector [15]. This wide range of support is commendable however, ongoing support for the strategy by the UK government will be essential to achieve the target of an average daily salt intake of 6 grams by 2012. Despite a government commitment in the recently published public health white paper to work with industry to achieve “further reformulation of food to reduce salt” [33] it is of concern that responsibility for nutrition policy was removed from the independent Food Standards Agency in October 2010 [34]. Mandatory targets for industry to reduce salt in processed foods and mandatory food labelling should be considered if more rapid progress toward the 6 grams per day target is not achieved. Evaluation of the Finnish salt reduction strategy, which was implemented in 1979 and had voluntary agreements with the food industry, suggest that average daily salt intake decreased by only 3 grams per day over a 20 year period (from 12 grams per day in 1979 to 9 grams per day in 2002) [35]. Our finding that the public awareness campaign resulted in only small reductions in voluntary use of salt in food preparation further highlight the importance of working with to industry to achieve large reductions in the salt content of processed foods. While we were unable to determine the relative contribution of the Food Standards Agency's public awareness campaign and reformulation of processed foods by industry to the reductions in salt intake identified, we would agree with calls for comprehensive salt reduction policies with a multi-pronged approach. Sodium reduction strategies need to be tailored to individual country contexts, including sources of salt within the diet, but may include communication strategies with the public; household level interventions, reformulation of foods; engagement with industry, possible regulation and ongoing monitoring of salt intake levels [36].

Policy makers in other countries should consider following the UK approach of designing and implementing salt reduction strategies in ways to bring disproportionate benefit to disadvantaged communities who experience higher rates of cardiovascular disease. Our findings suggest that persons from manual occupational groups and ethnic minorities achieved similar reductions in salt intake as non-manual and white ethnic groups. It is important that monitoring strategies for salt reduction strategies currently being developed by the World Health Organisation consider equity impacts explicitly in their evaluation frameworks [37].

## References

[1] Asaria P, Chisholm D, Mathers C, Ezzati M, Beaglehole R (2007). Chronic disease prevention: health effects and financial costs of strategies to reduce salt intake and control tobacco use.. The Lancet.

[2] Bibbins-Domingo K, Chertow GM, Coxson PG, Moran A, Lightwood JM (2010). Projected Effect of Dietary Salt Reductions on Future Cardiovascular Disease.. New England Journal of Medicine.

[3] Smith-Spangler CM, Juusola JL, Enns EA, Owens DK, Garber AM (2010). Population Strategies to Decrease Sodium Intake and the Burden of Cardiovascular Disease.. Annals of Internal Medicine.

[4] Joffres MR, Campbell NR, Manns B, Tu K (2007). Estimate of the benefits of a population-based reduction in dietary sodium additives on hypertension and its related health care costs in Canada.. The Canadian journal of cardiology.

[5] Mateo GF, O'Flaherty M, Lloyd-Williams F, Nnoaham K, Rayner M (2010). Estimating the UK cardiovascular mortality reduction expected with different food policy options.. Journal of Epidemiology and Community Health.

[6] Nishida C, Uauy R, Kumanyika S, Shetty P (2004). The Joint WHO/FAO Expert Consultation on diet, nutrition and the prevention of chronic diseases: process, product and policy implications.. Public Health Nutrition.

[7] He FJ, MacGregor GA (2008). A comprehensive review on salt and health and current experience of worldwide salt reduction programmes.. J Hum Hypertens.

[8] Food Standards Agency: Eat Well, Be Well.. http://www.eatwell.gov.uk/healthydiet/fss/salt/.

[9] Department of Health: Report of the Cardiovascular Review Group of the Committee on Medical Aspects of Food Policy (1994) (1994).

[10] Scientific Advisory Committee on Nutrition (2003). http://www.sacn.gov.uk/pdfs/sacn_salt_final.pdf.

[11] Capewell S, Graham H (2010). Will Cardiovascular Disease Prevention Widen Health Inequalities?. PLoS Med.

[12] Frohlich KL, Potvin L (2008). Transcending the Known in Public Health Practice: The Inequality Paradox: The Population Approach and Vulnerable Populations.. Am J Public Health.

[13] Health Survey for England. Chapter 5 (2007). Adult diet and healthy eating.. http://www.ic.nhs.uk/webfiles/publications/HSE07/HSE%2007-Volume%201.pdf.

[14] The National Diet and Nutrition Survey. Vitamin and mineral intake and urinary analytes.. http://www.food.gov.uk/multimedia/pdfs/ndnsv3.pdf.

[15] Food Standards Agency – UK salt reduction initiatives.. http://www.food.gov.uk/multimedia/pdfs/saltreductioninitiatives.pdf.

[16] Vollmer WM, Sacks FM, Ard J, Appel LJ, Bray GA (2001). Effects of Diet and Sodium Intake on Blood Pressure: Subgroup Analysis of the DASH-Sodium Trial.. Annals of Internal Medicine.

[17] Erlinger TP, Vollmer WM, Svetkey LP, Appel LJ (2003). The potential impact of nonpharmacologic population-wide blood pressure reduction on coronary heart disease events: pronounced benefits in African-Americans and hypertensives.. Preventive Medicine.

[18] Health Survey for England (2007). Volume 2 Methodology and Documentation.. http://www.ic.nhs.uk/webfiles/publications/HSE07/HSE%2007-Volume%202.pdf.

[19] Health Survey for England (2009). Volume 2: Methods and documentation.. http://www.ic.nhs.uk/webfiles/publications/003_Health_Lifestyles/hse09report/HSE_09_Volume2.pdf.

[20] Health Survey for England (2006). Latest Trends.. http://www.ic.nhs.uk/pubs/hse06trends.

[21] National Centre for Social Research (2008). An assessment of dietary sodium levels among adults (aged 19–64) in the UK general population in 2008, based on analysis of dietary sodium in 24 hour urine samples.. http://www.food.gov.uk/multimedia/pdfs/08sodiumreport.pdf.

[22] Bennett S (1995). Cardiovascular Risk Factors in Australia: Trends in Socioeconomic Inequalities.. Journal of Epidemiology and Community Health (1979-).

[23] World Health Organisation. Sodium intakes around the world.. http://www.who.int/dietphysicalactivity/Elliot-brown-2007.pdf.

[24] The Federation of Bakers - Salt.. http://www.bakersfederation.org.uk/currentissue.aspx?id=25.

[25] Association of Cereal Food Manufacturers - News.. http://www.breakfastcereal.org/news_slashsalt.html.

[26] Wyness LA, Butriss JL, Stanner SA (2011). Reducing the population's sodium intake: the UK Food Standards Agency's salt reduction programme.. Public Health Nutrition FirstView.

[27] The National Diet and Nutrition Survey. http://www.dh.gov.uk/prod_consum_dh/groups/dh_digitalassets/documents/digitalasset/dh_128542.pdf.

[28] National Centre for Social Research (2007). A survey of 24 hour and spot urinary sodium and potassium excretion in a representative sample of the Scottish population.. http://www.food.gov.uk/multimedia/pdfs/scotlandsodiumreport.pdf.

[29] Primatesta P, Poulter NR (2006). Improvement in hypertension management in England: results from the Health Survey for England 2003.. J Hypertens.

[30] Ashworth M, Medina J, Morgan M (2008). Effect of social deprivation on blood pressure monitoring and control in England: a survey of data from the quality and outcomes framework.. BMJ.

[31] Falaschetti E, Chaudhury M, Mindell J, Poulter N (2009). Continued Improvement in Hypertension Management in England.. Hypertension.

[32] (2008).

[33] Department of Health (2010). Healthy Lives, Healthy People. Our strategy for public health in England.. http://www.dh.gov.uk/prod_consum_dh/groups/dh_digitalassets/dh/en/ps/documents/digitalasset/dh_122347.pdf.

[34] Foods Standards Agency. Healthier eating.. http://www.food.gov.uk/healthiereating/.

[35] Laatikainen T, Pietinen P, Valsta L, Sundvall J, Reinivuo H (2006). Sodium in the Finnish diet: 20-year trends in urinary sodium excretion among the adult population.. Eur J Clin Nutr.

[36] Cappuccio FP, Capewell S, Lincoln P, McPherson K (2011). Policy options to reduce population salt intake.. BMJ.

[37] World Health Organisation. Population sodium reduction strategies.. http://www.who.int/dietphysicalactivity/reducingsalt/en/.

